# Role of Anti-Tuberculous Treatment in the Outcome of Decortication for Chronic Tuberculous Empyema

**DOI:** 10.7759/cureus.12583

**Published:** 2021-01-08

**Authors:** Nazish Sikander, Tanveer Ahmad, Misauq Mazcuri, Nadir Ali, Pratikshya Thapaliya, Shagufta Nasreen, Ambreen Abid

**Affiliations:** 1 Thoracic Surgery, Jinnah Postgraduate Medical Centre, Karachi, PAK

**Keywords:** chronic tuberculous empyema, decortication, anti-tuberculous therapy, lung expansion, air leak, outcome

## Abstract

Introduction: Chronic tuberculous empyema (CTE) is a common complication of tuberculosis that requires some form of surgical intervention along with anti-tuberculosis therapy (ATT). The aim of this study was to determine the optimum duration of pre-operative ATT in CTE prior to the decortication and its outcomes.

Material and Methods: This comparative prospective study was conducted from August 2019 to August 2020 in the Department of Thoracic Surgery, Jinnah Postgraduate Medical Centre, Karachi, Pakistan. A total of 70 patients were included in the study. They were grouped into two arms: patients operated at or within six weeks of ATT commencement (Group A) and patients operated after six weeks of ATT (Group B). Both groups had 35 participants each. Patients were evaluated based on a self-administered questionnaire. A p-value of less than 0.05 was considered significant.

Result: In this study, there were 55 (78.6%) males and 15 (21.4%) females with a mean age of 33.5 ± 11.2 years. Diagnosis of CTE was most commonly made through sputum acid-fast bacilli (AFB) smear (n=35, 50%) which most commonly involved right upper (n=20, 28.6%) and lower lung lobes (n=20, 28.6%). Complications such as air leaks, need for ventilator support, need for intensive care unit (ICU) stay, residual collection, and pneumothorax all were significantly higher in Group A (31 patients out of 35) compared to Group B (18 patients out of 35). In Group B, 21 (60%) participants had full post-operative expansion of lungs, compared to eight (22.8%) in Group A (p=0.002). In total five participants had failure to expand lungs; all of them belonged to Group A (p=0.02).

Conclusion: The optimum timing of surgery and preoperative ATT is crucial for achieving better outcomes and requires good collaboration between the treating pulmonologist and thoracic surgeon. Our study highlights the importance of pre-operative ATT for at least more than six weeks before undertaking decortication for better outcomes and minimizing morbidity.

## Introduction

Empyema thoracis is a pyogenic infection of an otherwise sterile pleural cavity characterized by accumulation of pus [1]. It is classified into three stages according to the American Thoracic Society: exudative, fibrinopurulent, and organizing [2]. It remains a common clinical problem in both developed and developing countries, however the etiology differs. Pneumonia and thoracic trauma are the usual causes in the population of the developed world while tuberculosis accounts for most cases in the latter [3]. It affects approximately 65,000 patients per year in developed countries [4]. However, the incidence is quite high in developing countries; 265 cases per 100,000 in Pakistan according to the World Health Global Tuberculosis report 2019 [5].

Chronic tuberculous empyema (CTE) is a late complication of tuberculous lung infection [6]. It cannot be treated with anti-tuberculous drugs alone and needs surgical evacuation of the pus. Surgical techniques range from tube thoracostomy, intrapleural fibrinolytics, open window thoracostomy to thoracotomy and decortication [7,8]. These surgical techniques are recommended in conjunction with chemotherapy like anti-tuberculosis therapy (ATT) in cases of CTE. ATT is recommended for at least six weeks prior to decortication [9]. However, some studies have reported a range of two weeks to 12 months of ATT before decortication surgery [10]. Despite numerous studies, there is considerable variation in timing the surgery for CTE and it is still controversial [11]. An established treatment strategy will probably give the best outcome of decortication with complete expansion of lung, thus preventing morbidity. In this study, we aim to determine the optimum duration of pre-operative ATT in CTE prior to decortication and its outcomes.

## Materials and methods

This comparative prospective study was conducted in the Department of Thoracic Surgery, Jinnah Postgraduate Medical Centre, Karachi from August 2019 to August 2020. The Institutional Review Board Committee issued approval No.F.2-81/2019-GENL/36837/JPMC. Inclusion criteria were patients of both genders, age more than 12 years, with diagnosis of tuberculous empyema on clinical and radiological grounds, fit for surgery, and body mass index greater than 18 kg/m^2^. All patients were on ATT prior to any major surgical intervention. Exclusion criteria included empyema due to other causes, body mass index less than 18 kg/m^2^, multi-drug resistant tuberculosis, patients with cardiac comorbidity, intraparenchymal tuberculous infection, and pulmonary fibrosis.

Patients were divided into two groups on the basis of duration of ATT. Group A were operated at or within six weeks of ATT commencement, while Group B were operated after six weeks of ATT. Both groups had 35 participants each.

The indications for decortication were trapped lung due to thickened pleural cortex, empyema with or without bronchopleural fistula (BPF), disease progression or relapse despite ATT, and symptomatic bronchiectasis. Patient informed consent was taken. A double lumen endotracheal tube was used for the induction of anesthesia followed by placing the patient in lateral decubitus position. Posterolateral thoracotomy incision was utilized with resection of the fifth rib. Mobilization of pleura was undertaken with evacuation of all purulent material. Decortication was performed with the goal of complete lung expansion. A large-bore chest tube was inserted and attached to an underwater seal at the end of the operation. Demographics of patients and outcome of surgery were noted in a self-structured questionnaire. Outcomes were defined according to postoperative lung expansion. Full lung expansion characterized as radiographic evidence of fully expanded lung and absence of residual collection or pleural thickening on at least two postoperative chest X-ray films. Partial lung expansion was defined as visibly collapsed lung border with or without blunting of cardiophrenic or costophrenic angle with or without a fluid meniscus on chest radiograph after removal of chest tube within the same admission [12]. Failure of lung expansion was characterized as no change in the expansion of lung noted in the postoperative radiograph when compared with preoperative imaging.

Numerical data such as chest tube duration, in-hospital stay, weight, duration of symptoms were recorded as mean and standard deviation (SD). Categorical data such as complications and outcomes were measured as frequencies and percentages. Means were compared using t-test. Categorical data were compared using Chi-square. A p-value of less than 0.05 indicates that the difference between Group A and Group B is significant enough to discard the null hypothesis.

## Results

In this study, there were 55 (78.6%) males and 15 (21.4%) females with a mean age of 33.5 ± 11.2 years. The most common side for tuberculosis was right side. History of contact with tuberculosis patients was positive in 36 (60.0%) patients. Diagnosis was most commonly made through the sputum for AFB test (50%). Right upper lobe [n=20 (28.6%)] and right lower lobe [n=20 (28.6%)] were the most affected lobes followed by left upper lobe in 15 (21.4%) participants. Chest tube was inserted in 54 (77.1%) participants when admitted. Hydropneumothorax at the time of diagnosis was present in 49 (70%) patients. Fistula was present in 46 (65.7%) patients. Chronic obstructive pulmonary disease was found in 20 (28.6%) participants whereas 14 (20%) had hepatitis C. Mean hospital stay was 15.5 ± 7.6 days. Length of hospitalization significantly differed among both groups (p value= 0.018). Table 1 demonstrates stratification of patients.

**Table 1:** Stratification of patients on basis of demography, disease (N=70).

Characteristics	N (%)
Gender	
Male	55 (78.6%)
Female	15 (21.4%)
Side	
Right	42 (60%)
Left	28 (40%)
Diagnosis by	
Sputum Acid Fast Bacillus (AFB)	35 (50%)
Clinical/radiological	23 (32.8%)
Sputum gene xpert	8 (11.4%)
Pleural Fluid AFB	3 (4.3%)
Pleural fluid gene xpert	1 (1.4%)
Lobes Affected	
Right Upper lobe	20 (28.6%)
Right lower lobe	20 (28.6%)
Left Upper Lobe	15 (21.4%)
Left Lower Lobe	13 (18.6%)
Right Middle Lobe	2 (2.9%)
Air Leak	
No air leak	22 (31.4%)
On forced expiration (Grade I)	15 (21.4%)
On expiration only (Grade II)	25 (35.7%)
On inspiration only (Grade III)	8 (11.4%)
Continuous air leak (Grade IV)	0
Noncompliance of Anti-tuberculosis therapy	
No	39 (55.7%)
Yes	31(44.3%)
Additional Procedure Performed	
None	35 (50%)
Wedge	18 (25.7%)
Lobectomy	17(24.3%)

Complications were significantly higher in Group A compared to Group B (0.003). Air leaks, need for ventilatory support, need for Intensive Care Unit (ICU) stay, residual collection, and pneumothorax all were significantly higher in Group A compared to Group B (Table 2).

**Table 2:** Complications in both groups.

Complications		Group A (n=35)	Group B (n=35)	P value
Complications	Yes	31	18	0.001
	No	4	17	
Air Leaks	Less than 5 days	13	11	0.001
	More than 5 days	18	7	
	None	4	17	
Ventilator Support	Yes	10	3	0.031
	No	25	32	
Intensive Care Unit (ICU) stay	Yes	30	18	0.002
	No	5	17	
Second Tube Inserted	Yes	8	3	0.101
	No	27	32	
Residual Collection	Yes	27	14	0.002
	No	8	21	
Residual Pneumothorax	Yes	23	9	0.001
	No	12	26	

Patients with residual collection were subjected to ultrasound-guided aspiration, however few of them needed placement of a second tube. Patients with residual pneumothorax were discharged with Heimlich valve 9®, called for follow-up weekly and the tube was removed after two weeks.

In Group B, 21 (60%) participants had full post-operative expansion of lungs, compared to eight (22.8%) in Group A. In total five participants had failure to expand lungs; all of them belonged to Group A (Table 3).

**Table 3:** Outcome in terms of lung expansion in both groups.

Post-Operative Outcome		Group A (n=35)	Group B (n=35)	P value
Full Lung Expansion	Yes	8	21	0.002
	No	27	14	
Partial Lung Expansion	Yes	22	14	0.05
	No	13	21	
Failure to Expand	Yes	5	0	0.02
	No	30	35	

## Discussion

Chronic tuberculous empyema is the late sequelae of pleuropulmonary tuberculosis as a consequence of rupture of the subpleural lesion, nodal or hematogenous spread of the primary pulmonary disease to the pleural space [13]. It remains a cause of morbidity and mortality in developing countries [11]. According to the World Health Organization Global Tuberculosis Report 2019, Pakistan is the eighth highest tuberculosis (TB) burden countries with an incidence of 265 cases per 100,000 population [5].

CTE affects people of all ages in both genders but is found to be more common in males [11], a finding consistent with our study as well (males n=55, 78.6%). Diagnosis can be made by acid-fast bacilli (AFB) smear and gene expert of sputum or pleural fluid, radiological or pleural biopsy and culture [14]. The major diagnostic tool in our study was sputum AFB smear (50%) followed by radiological evidence (32.8%). The prevalence of hepatitis C in our study was 20% (n=14) as opposed to 7% in a meta-analysis, thus increasing the chance of hepatotoxicity due to ATT [15]. All cases in our study had unilateral involvement with the majority being right-sided (n=42, 60%), as stated in another study, however, the side of involvement does not affect the prognosis [16]. 

Bronchopleural fistula (BPF) occurs 2.74 times more frequently in patients with empyema. A local study conducted at a tertiary care hospital revealed the presence of BPF in 31.6% of empyema patients whereas we found BPF in 65.7% cases [17].

The management of empyema is stage-dependent. Stage I (Exudative) and Stage II (Fibrinopurulent) can be managed conservatively with antibiotics and drainage[13]. Stage III (Organizing) is characterized by formation of granulation tissue and entrapment of the lung by a thick cortex, warranting invasive approach [2]. Management of CTE is challenging because of extensive pleural thickening, contracted chest cavity, and poor nutritional status [13]. It requires multimodal therapy with ATT, tube thoracostomy, nutritional build-up, and ultimately decortication for drainage of residual purulent fluid, thus reducing septic load and removal of pleura and allowing lung expansion [13,18]. Pre-operative ATT has a significant role despite acquired drug resistance due to different penetration of drugs through the fibrocalcific cortex [18,19]. It carries a significant risk of reactivation of tuberculosis if left untreated [19]. Thus high-end dosing of ATT is recommended pre-operatively followed by its recommencement postoperatively for a sufficient period to eliminate the residual disease [18,19].

The indications of decortication include entrapped lung, calcified pleura with >2cm thickness, crowding of ribs, empyema with or without BPF, disease progression despite ATT, symptomatic bronchiectasis, and high chances of relapse with medical management [13,18]. Similar indications were included in our study. Complications of decortication comprise prolonged air leak (PAL), residual collection, residual pneumothorax, and mechanical ventilation; consistent with our study as well [20].

PAL is defined as an air leak that persists beyond five days postoperatively and the frequency ranges between 18 to 58% according to one study [21]. We found that the occurrence of PAL was higher in Group A (51.4%) as compared to 20% in Group B (p=0.001). This can be explained by inadequate ATT and failure of disease clearance.

Persistent pleural space and significant air leak after decortication leads to complications such as persistent pneumothorax and residual collection [21,22]. In another study, it was reported an incidence of persistent residual air space after surgery for tuberculosis as high as 21% to 33% [21]. In our study, we found the occurrence of residual collection (p=0.002) and persistent pneumothorax (p=0.001) significantly higher in Group A as compared to Group B.

Another cohort study elaborated that an infectious etiology leading to decortication was associated with a higher risk of mechanical ventilation of around 18% due to diseased lung parenchyma [20]. It may also be attributed to the release of loculated infection, host inflammatory response, and physiological stress of anesthesia and decortication in an already vulnerable population [20]. In our study, Group A candidates required mechanical ventilation in 28.5% of cases whereas Group B required it in only 8.5% of patients (p=0.03).

Complete lung re-expansion and pleural apposition is the ultimate goal of decortication [20]. It was achieved in 60% of participants of Group B (n=21, p=0.002) compared to 22.8% (n=8) in Group A in our study. Okiror and colleagues emphasized that patients with culture-positive pleural infection and inadequate preoperative antibiotics are more prone to complications along with higher rates of failure of surgery characterized by persistent pleural space/effusion or trapped lung. They found 28% of participants with partial lung expansion after decortication [12]. However, in our study partial lung expansion was the fate of 22 participants of Group A as compared to 14 in Group B (p=0.05). Five participants had failure of lung expansion and all of them belonged to Group A (p=0.02).

Thus, the optimum timing of surgery and preoperative ATT is crucial for achieving better outcomes and requires good collaboration between the treating pulmonologist and thoracic surgeon [18]. Our study highlights the importance of pre-operative ATT for at least more than six weeks before undertaking decortication for better outcomes and minimizing morbidity. This study reports data from the largest tertiary care institute in the metropolitan city of Pakistan. However, it has its limitations too. It represents the prevalence of disease from a single institute only. Thus, the data cannot be generalized. However, it does lay the ground for future research in this subject.

## Conclusions

Chronic tuberculous empyema is one of the commonest reasons for morbidity and mortality in endemic regions of the world. Surgical interventions are the mainstay for evacuation of such empyemas. The duration of anti-tuberculous therapy is crucial for determining the better outcome of surgery. This study suggests that consumption of anti-tuberculous therapy for more than six weeks prior to surgery favors better outcome in terms of lung expansion, morbidity, and early recovery.
